# Supplementary material for: Active site specificity profiling datasets of matrix metalloproteinases (MMPs) 1, 2, 3, 7, 8, 9, 12, 13 and 14
Source: Data Brief. 2016 Feb 22;7:299–310. doi: 10.1016/j.dib.2016.02.036 (PMC4777984; doi:10.1016/j.dib.2016.02.036)
Supplement: Supplementary file 10 — Supplementary material [file mmc10.zip › WebPICS_hMMP13_T_1%/P3.html]

 

PICS results


|  |  |
| --- | --- |
| **P3\_A**  19 in 143 sites   13.3 %    effects > 10 perc. pnts.  (vice-versa in brackets)  P1\_N: 10.6 (13.4)   P3prime\_N: 10.2 (24.2) |  |
| **P3\_I**  11 in 143 sites   7.7 %    effects > 10 perc. pnts.  (vice-versa in brackets)  P1\_H: 23.8 (52.3)   P1prime\_C: 12.6 (17.3) |  |
| **P3\_P**  58 in 143 sites   40.6 %    effects > 10 perc. pnts.  (vice-versa in brackets)  P1prime\_L: -11.1 (-15.0) |  |
| **P3\_V**  13 in 143 sites   9.1 %    effects > 10 perc. pnts.  (vice-versa in brackets)  P1prime\_M: 11.2 (24.2) |  |
